# Intranasal Immunization With a c-di-GMP-Adjuvanted Acellular Pertussis Vaccine Provides Superior Immunity Against *Bordetella pertussis* in a Mouse Model

**DOI:** 10.3389/fimmu.2022.878832

**Published:** 2022-04-13

**Authors:** Wenwen Jiang, Xiaoyu Wang, Yuhao Su, Lukui Cai, Jingyan Li, Jiangli Liang, Qin Gu, Mingbo Sun, Li Shi

**Affiliations:** ^1^Yunnan Key Laboratory of Vaccine Research and Development on Severe Infectious Diseases, Institute of Medical Biology, Chinese Academy of Medical Science & Peking Union Medical College, Kunming, China; ^2^Laboratory of Immunogenetics, Institute of Medical Biology, Chinese Academy of Medical Science & Peking Union Medical College, Kunming, China; ^3^Laboratory of Vaccine Development, Institute of Medical Biology, Chinese Academy of Medical Science & Peking Union Medical College, Kunming, China

**Keywords:** *Bordetella pertussis*, acellular pertussis vaccine (aP), c-di-GMP, 2’,3’-cGAMP, cyclic dinucleotides (CDNs), mucosal immunity

## Abstract

Pertussis, caused by the gram-negative bacterium *Bordetella pertussis*, is a highly contagious respiratory disease. Intranasal vaccination is an ideal strategy to prevent pertussis, as the nasal mucosa represents the first-line barrier to *B. pertussis* infection. The current intramuscular acellular pertussis (aP) vaccines elicit strong antibody and Th2-biased responses but not necessary cellular and mucosal immunity. Here, we formulated two cyclic dinucleotide (CDN)-adjuvanted aP subunit vaccines, a mammalian 2’,3’-cGAMP-adjuvanted aP vaccine and a bacterial-derived c-di-GMP-adjuvanted aP vaccine, and evaluated their immunogenicity in a mouse model. We found that the aP vaccine alone delivered intranasally (IN) induced moderate systemic and mucosal humoral immunity but weak cellular immunity, whereas the alum-adjuvanted aP vaccine administered intraperitoneally elicited higher Th2 and systemic humoral immune responses but weaker Th1 and Th17 and mucosal immune responses. In contrast, both CDN-adjuvanted aP vaccines administered *via* the IN route induced robust humoral and cellular immunity systemically and mucosally. Furthermore, the c-di-GMP-adjuvanted aP vaccine generated better antibody production and stronger Th1 and Th17 responses than the 2′,3′-cGAMP-adjuvanted aP vaccine. In addition, following *B. pertussis* challenge, the group of mice that received IN immunization with the c-di-GMP-adjuvanted aP vaccine showed better protection than all other groups of vaccinated mice, with decreased inflammatory cell infiltration in the lung and reduced bacterial burden in both the upper and lower respiratory tracts. In summary, the c-di-GMP-adjuvanted aP vaccine can elicit a multifaceted potent immune response resulting in robust bacterial clearance in the respiratory tract, which indicates that c-di-GMP can serve as a potential mucosal adjuvant for the pertussis vaccine.

## Introduction

Pertussis is a highly infectious respiratory disease caused by *Bordetella pertussis (B. pertussis)* and remains a lethal threat in unvaccinated infants. Vaccination is the most effective way to prevent pertussis. To date, acellular pertussis (aP) vaccines have gradually replaced whole pertussis (wP) vaccines due to their fewer adverse side effects (1, 2). However, recent epidemiological data show that pertussis has experienced a resurgence in several countries, even in countries with nearly universal vaccine coverage, in the last 20 years (3, 4). Many hypotheses have been proposed to explain the pertussis resurgence, including increased detection sensitivity, vaccine-driven evolution of *B. pertussis* strains, waning of protective efficacy of aP vaccines, and asymptomatic transmission (5–7). Among all these reasons, the inefficient protection afforded by current aP vaccines may be the major issue for the insufficient prevention and control of whooping cough. Several studies have suggested that current aP vaccines cannot prevent *B. pertussi*s infection and transmission because they induce only humoral immune responses but not efficient cellular and mucosal immune responses (8–10). The current intramuscular acellular pertussis (aP) vaccines elicit strong antibody and Th2-biased responses but not necessary cellular and mucosal immunity. Th1 cells-mediated immune responses is generally considered to be cellular or cell-mediated immunity (CMI), while Th2 cells can provide optimal help for humoral immune responses (11). Since routine aP vaccines are formulated with several pertussis components with an aluminum adjuvant, administered *via* intramuscular injection and mainly induce antibody protection, novel aP vaccines with appropriate adjuvants administered *via* intranasal (IN) inoculation have been a research hotspot.

The upper respiratory tract (URT) is the site of infection for *B. pertussis*, and the pre-existing immunity on mucosal surfaces of the respiratory tract plays a crucial role in defense against *B. pertussis* infection (12). Studies on nonhuman primates have shown that potent local humoral and cellular immune responses, especially Th17 responses, induced by natural *B. pertussis* infection can provide complete protection against reinfection (8, 13). In addition, many studies have shown that the IN administration of aP vaccines combined with an appropriate adjuvant induced optimal protection against infection, especially in the URT (14–16). Therefore, mucosal immunity might offer a crucial mechanism to prevent nasal colonization and infection.

Cyclic dinucleotides (CDNs), which can trigger the innate immune response in mammalian cells *via* the stimulator of interferon genes (STING) signaling pathway, leading to type I interferon (IFN) generation, have been studied as novel vaccine adjuvants (17). Many studies have shown that CDNs have strong mucosal adjuvant properties (18–21). CDNs, consisting of two nucleotide residues linked by two phosphodiester bonds, have been recognized as a class of crucial secondary signaling molecules in bacteria and in mammalian cells, with robust immunomodulatory and immunostimulatory functions. Depending on the pair of phosphodiester linkages, CDNs have several isomers. There are four common CDNs, three in bacteria (c-di-GMP, c-di-AMP, and 3’,3’-cGAMP) and one in eukaryotic cells (2’,3’-cGAMP), and the bacterial Sting pathway, which plays an important role in the defense against bacteriophages, prefers canonical 3’–5’-linked CDNs (22). c-di-GMP is a universal bacterial secondary messenger in gram-negative bacteria and is defined as two GMP molecules linked *via* two 3′-5′ phosphodiester bonds (22, 23). c-di-GMP participates in many bacterial processes, including virulence, stress survival, motility, metabolism, antibiotic production, differentiation, biofilm formation, and other processes (24). Recent research has shown that c-di-GMP, acting as a danger signal in eukaryotic cells, is recognized by mammalian immune systems and therefore is considered a potential vaccine adjuvant (25). Another CDN, 2’,3’-cGAMP, containing mixed phosphodiester linkages connecting the two nucleosides from the 2 and 5 positions of guanosine and the 3 and 5 positions of adenosine, is synthesized by cGAS from ATP and GTP upon cytosolic DNA stimulation and was first discovered in 2012 (26, 27). This mammalian CDN isomer is different from all characterized bacterial CDNs. Eukaryotic cells employ a phosphodiester linkage (2′-5′) to promote greater CDN stability, thus allowing stronger and more prolonged signal amplification. In addition, the unique 2′-5′- phosphodiester linkage might be a defense mechanism of eukaryotic cells that allows them to avoid subversion of the innate immune response by bacteria because bacterial cells might not be able to degrade 2′-5′ phosphodiester linkages (22). As seen with other CDNs, 2′,3′-cGAMP also has potential applications as an adjuvant (28). All CDNs can bind and stimulate the STING signaling pathway in eukaryotic cells, but 2′,3′-cGAMP binds to mammalian sting with a much greater affinity than bacterial CDNs because of their different phosphodiester linkage positions (27, 29). Moreover, 2′,3′-cGAMP induces stronger type I IFN production than other CDNs derived from bacteria (27). Thus, we selected 2′,3′-cGAMP and c-di-GMP as adjuvants to determine which could provide a superior mucosal immune response against *B. pertussis*.

In this study, we compared and evaluated the efficacy of two common CDNs *in vitro* by using bone marrow-derived dendritic cells (BMDCs). Subsequently, we formulated the test aP vaccine containing pertussis toxoid (PT), filamentous hemagglutinin (FHA), and pertactin (PRN) using the two types of CDNs as adjuvants. We examined the two test aP vaccine-induced immune responses and protective efficacy against *B. pertussis* in a mouse model to evaluate whether the 2′,3′-cGAMP- or c-di-GMP-adjuvanted aP vaccine could be a candidate vaccine against *B. pertussis*.

## Materials and Methods

### Mice and Ethics Statements

Specific pathogen-free (SPF) 4- to 5-week-old male and female BALB/c mice were purchased from Beijing Charles River Laboratory (Beijing, China). All mice used in this study were treated in accordance with the Guide for the Care and Use of Laboratory Animals of the People’s Republic of China. All protocols were reviewed and approved by the Committee on Ethics of the Institute of Medical Biology, Chinese Academy of Medical Sciences (IMBCAMS; assurance number: DWSP202106004). Animals were bred and maintained under SPF conditions at IMBCAMS at a constant temperature (20–24 °C) and humidity (45–65%), with lighting on a fixed light/dark cycle (12 h/12 h).

### Bacterial Strains, Media, and Growth Conditions

The *B. pertussis* strain *B.p-L1* used in this study was recently isolated from a patient in Yunnan Province, China. Total DNA was extracted from the recovered *B. pertussis*, which was characterized as carrying the ptxP3 genotype by DNA sequencing of the pertussis toxin promoter (ptxP) (30). For *B. pertussis* infection experiments, bacteria were grown on Bordet-Gengou agar (B-G) plates (Hopebio) containing 20% defibrinated sheep blood (Nanjinglezhen) for 24 to 48 h at 37°C. Colonies from fresh B-G plates were resuspended in phosphate-buffered solution (PBS), diluted to a concentration of 10^11^ CFU/mL by using a turbidimetric method, and used within 2 h of preparation. For culture of bacteria from tissues, Regan-Lowe plates prepared from Regan-Lowe charcoal agar base (Oxoid) supplemented with 10% defibrinated sheep blood and 40 μg/mL cephalexin (Oxoid) were used.

### Isolation and Stimulation of Dendritic Cells

BMDCs were isolated from mouse bone marrow cells as previously described (31). Briefly, bone marrow cells were isolated and cultured in RPMI 1640 medium containing 20 ng/mL recombinant GM-CSF (Peprotech), 100 U/mL penicillin, 100 µg/mL streptomycin, and 10% FBS. Petri dishes containing 2 × 10^6^ cells in 10 mL were incubated at 37°C in 5% CO_2_. At day 3 of incubation, an additional 10 mL of fresh complete medium containing GM-CSF was added, and 10 mL of medium was replaced with fresh medium supplemented with GM-CSF on day 6. Immature BMDCs were collected on day 7 for experiments. The BMDCs were treated with 2′,3′-cGAMP (5 μg/mL, *In vivo*Gen, California, USA) or c-di-GMP (5 μg/mL, *In vivo*Gen, California, USA) *in vitro* for 24 h. Cells were treated with lipopolysaccharide (LPS, 1 μg/mL, Sigma–Aldrich) as a positive control or sterile PBS as a negative control. Cytokine (IFN-β and TNF-α) levels in the supernatant were quantified by ELISA. The stimulated BMDCs were stained with the specific antibodies described below and analyzed by using FlowJo software.

### Mouse Immunization and Sample Collection

**Figure 1:**
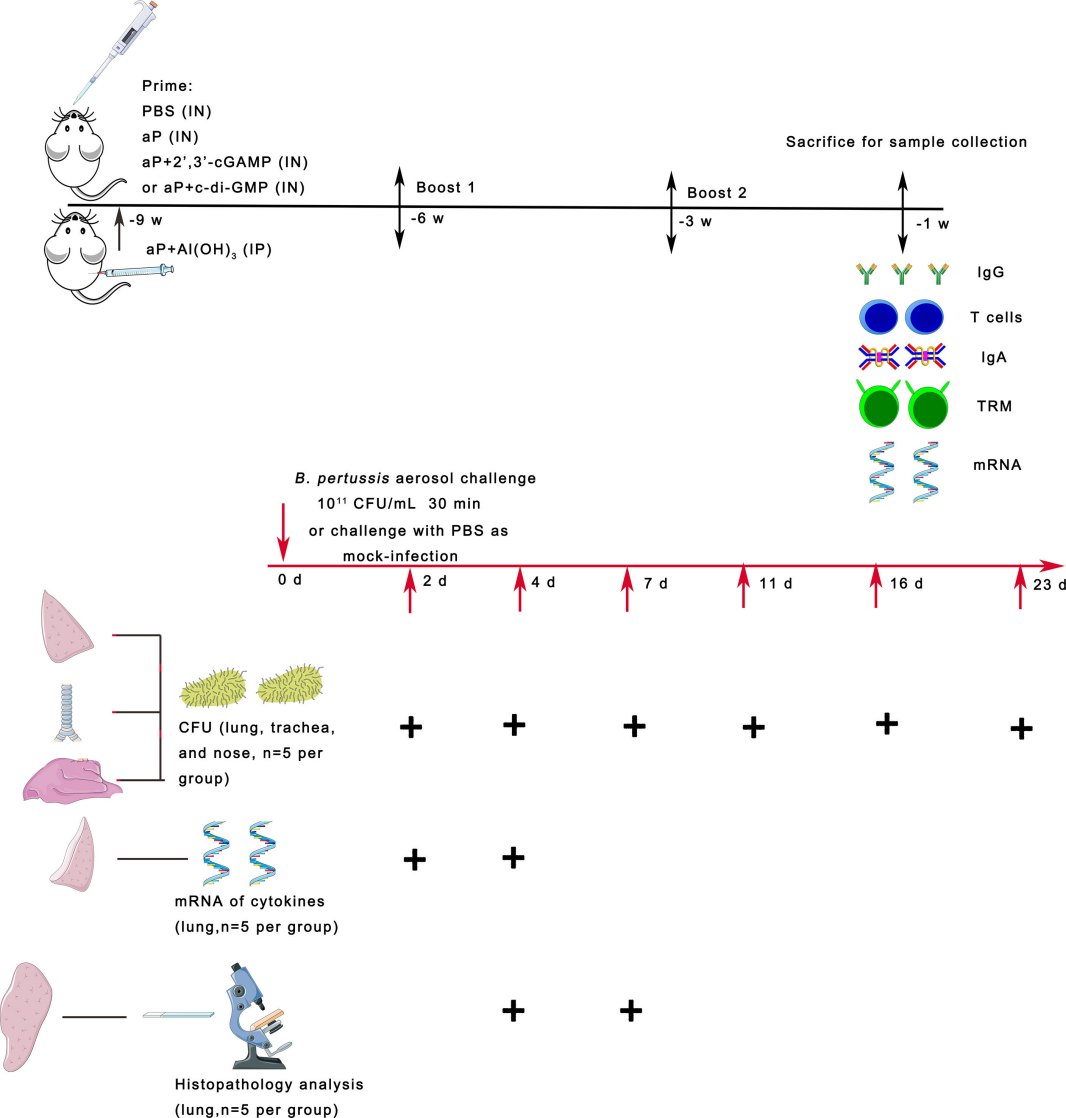
Scheme of mouse immunization and *B. pertussis* challenge. BALB/c mice were immunized with three doses of the different vaccines *via* the intranasal (IN) or intraperitoneal (IP) route at 3-week intervals. Control mice received PBS *via* the IN route. To evaluate specific immune responses, including systemic and mucosal immune responses, mice were bled and sacrificed two weeks after the last immunization for sample collection. Three weeks after the last immunization, the mice were infected with *B. pertussis* by aerosol challenge. In parallel, naïve mice were challenged with PBS as a mock-infection group. On days 2, 4, 7, 11, 16, and 23 after *B. pertussis* infection (dpi), lungs, trachea, and nasal mucosa were collected from each group of mice, and the numbers of *B. pertussis* CFU in the upper and lower respiratory tracts were determined by dilution plating. On days 2 and 4 after infection, the mRNA expression levels of the indicated cytokines were determined by real-time quantitative PCR (RT–qPCR) in the lung tissues. On days 4 and 7 after infection, lung tissues were collected, processed into paraffin sections and stained with H&E.

BALB/c mice (half of which were male and half of which were female, 4–5 weeks old) were immunized *via* the IN (20 μL volume) or intraperitoneal (IP, 200 μl volume) route three times at three-week intervals with the different tested vaccines (**Table 1**). Antigens of the aP vaccine were produced by the IMBCAMS under good manufacturing practice conditions (32). All vaccinations were performed under anesthesia (isoflurane). Blood, nasal washes (NWs), and bronchoalveolar lavage fluid (BALF) were collected two weeks after the last immunization. Blood was collected and centrifuged at 860 g for 10 min to obtain plasma. NW and BALF samples were obtained by washing the nasal cavity and lung with 0.2 mL and 1 ml of cold PBS containing protease inhibitor, respectively. Nasal cavity and lung wash fluids were centrifuged at 2400 g for 10 min, and the supernatants were collected. Plasma, NW, and BALF samples were stored frozen at -20°C until the detection of antibodies (**Figure 1**).

**Table 1:** The detail of immunization regimens.

Vaccine components (μg/dose)	PBS/IN	aP/IN	aP + Al(OH)_3_/IP	aP + 2’,3’-cGAMP/IN	aP + c-di-GMP/IN
PT	–	1.25	1.25	1.25	1.25
FHA	–	1.25	1.25	1.25	1.25
PRN	–	0.4	0.4	0.4	0.4
Al(OH)_3_	–	–	37.5	–	–
2’,3’-cGAMP	–	–	–	5	–
c-di-GMP	–	–	–	–	5

“IN” and “IP” indicate intranasally and intraperitoneally immunized, respectively.

“PT”, “FHA”, and “PRN” indicate pertussis toxoid, filamentous hemagglutinin, and pertactin.

### Preparation of Monocellular Suspensions From Organs

Spleen tissues were minced, filtered through a 70-μm nylon mesh (BD Biosciences), and diluted 1:1 in RPMI 1640. The mixture was loaded onto a Ficoll-Paque (GE, USA) layer and centrifuged at 1500 rpm for 30 minutes at room temperature. For monocellular isolation from lungs, tissues were minced and digested with collagenase D (1 mg/mL; Roche) and DNase I (20 U/mL; Roche) for 45 min at 37°C on a shaker. Next, tissues were passed through a 70-μm cell strainer, washed twice in complete RPMI 1640 medium, mixed with 40% Percoll, loaded onto a 70% Percoll layer and centrifuged at 1800 rpm for 30 minutes at room temperature. The cells obtained from organs were washed twice with RPMI 1640 and/or resuspended in complete cell culture medium (RPMI 1640 supplemented with 10% (V/V) fetal calf serum (FCS) and a 1% (v/v) premixed penicillin–streptomycin solution). The cells were used for cytokine detection, ELISpot, and/or flow cytometry.

### Enzyme-Linked Immune Sorbent Assay (ELISA)

Microplates (96-well) were coated with PT, FHA, or PRN at 3 μg/mL and incubated at 4°C overnight. Then, the plates were blocked with 3% (w/v) bovine serum albumin (BSA, Abcam) in PBS at 37°C for 2 h. Diluted serum, NW, or BALF sample was added to each microplate and incubated at 37°C for 1 h. After washing, a horseradish peroxidase (HRP)-labeled sheep anti-mouse IgG (Jackson ImmunoResearch, USA), anti-mouse IgG1 (Southern Biotech, USA), anti-IgG2a (Southern Biotech, USA), or anti-mouse IgA (Southern Biotech, USA) antibody was added to the microplate, and the plate was incubated at 37°C for 1 h. All the ELISA plates were developed using tetramethylbenzidine (TMB; Solarbio, CHN) to generate a colorimetric reaction, and the reaction was terminated with 2 mmol/L H_2_SO_4_. The absorbance of the plates at 450 nm was read. Endpoint titers were determined as the dilution that exhibited an optical density exceeding ≥2.1 times the background level (secondary antibody alone).

### Enzyme-Linked Immunospot (ELISpot)

T cell detection by IFN-γ, IL-4 and IL-17 ELISpot assays was performed according to the manufacturer’s instructions (Cellular Technology Limited, USA). Briefly, precoated 96-well plates were seeded with 5 μg/mL specific stimulants and 4 × 10^5^ mouse splenocytes or pneumonocytes in a total volume of 100 μL and incubated. Following a 24-h incubation at 37°C with 5% CO_2_, the cells were removed, and an anti-IFN-γ, anti-IL-4 or anti-IL-17 detection antibody was added, followed by the addition of streptavidin-ALP. For antigen-specific IgA or IgG plasma cell detection, MultiScreen filter 96-well plates (Millipore, USA) were precoated with PT, FHA, or PRN (each 5 μg) for each well overnight at 4°C. After rinsing with PBS, the plates were blocked with culture medium for 30 min at room temperature. Single-cell suspensions of splenocytes or pneumonocytes in culture medium were added to the coated plates and incubated at 37°C with 5% CO_2_ for 24 h. After washing with PBS, the plates were incubated with biotinylated anti-IgA or anti-IgG antibodies (Southern Biotech, USA) followed by incubation with streptavidin-conjugated horseradish peroxidase (Jackson ImmunoResearch, USA), each for 1 h at room temperature. After additional washes with PBS, AEC substrate solution (BD Bioscience, USA) was added for spot development. The reaction was stopped by rinsing with water. Spots were developed using BCIP/NBT and analyzed by a Cellular Technology Limited (CTL) ELISpot reader.

### ELISA for Cytokines

Cytokine levels in the supernatant of cultured cells were measured by an ELISA kit according to the manufacturer’s recommendation. Values were calculated based on a standard curve of recombinant cytokines. The results are expressed as picograms per milliliter (pg/mL). IFN-β (Cat# VAL612, Novus Biologicals) and TNF-α (Cat# VAL609, Novus Biologicals) levels in the supernatant of BMDCs were quantified. For detecting the cytokines level in the supernatant of cultured splenic or pulmonary lymphocytes, cells were cultured at a concentration of 2 × 10^6^/ml and stimulated with the antigens PT (2 μg/ml), FHA (2 μg/ml), and PRN (2 μg/ml). Supernatants were removed after 3 days and stored at -20°C before testing. IFN-γ (Cat# VAL607, Novus Biologicals), TNF-a (Cat# VAL609, Novus Biologicals), and IL-2 (Cat# VAL602, Novus Biologicals) levels were measured for Th1 responses; IL-5 (Cat# KA0253, Novus Biologicals) and IL-6 (Cat# VAL604, Novus Biologicals) levels were detected for Th2 responses; and IL-17A (Cat# VAL610, Novus Biologicals) and IL-22 (Cat# M2200, R&D Systems) levels were tested for Th17 responses (33, 34).

### Flow Cytometry

Single-cell suspensions were obtained from BMDCs or lung tissues. To detect BMDC maturation, cells were blocked with anti-mouse CD16/32 antibodies and stained for surface markers with anti-CD11c (Biolegend, clone: N418, Cat# 117338), anti-CD80 (Biolegend, clone: 16-10A1, Cat# 104729), anti-CD86 (eBioscience, clone: GL1, Cat# 25-0862-82), anti-CD40 (BD Bioscience, clone: 3/23, Cat# 562846), and anti- major histocompatibility complex molecule class II (MHC II) antibodies (I-A/I-E, eBioscience, clone: M5/114.15.2, Cat# 12-5321-8282). To discriminate circulating cells from lung-resident cells, intravascular staining was performed as previously described (31). In brief, mice were intravenously (i.v.) delivered with 3 μg of PE-labeled anti-CD45 antibody (eBioscience, clone: 30-F11, Cat# 12-0451-82) and sacrificed 10 min after i.v. injection, and lungs were isolated immediately to obtain a single-cell suspension as described. For the detection of lung tissue-resident memory T (TRM) cells, lung cells were stained for cell surface markers with anti-CD3 (Biolegend, clone: 17A2, Cat# 100216), anti-CD4 (Biolegend, clone: GK1.5, Cat# 100406), anti-CD8 (Biolegend, clone: 53-6.7, Cat# 100752), anti-CD44 (Biolegend, IM7, Cat# 103040), anti-CD69 (eBioscience, clone: H1.2F3, Cat# 25-0691-82), and anti-CD62L antibodies (Biolegend, clone: MEL-14, Cat# 104412). Dead cells were excluded by 7-AAD staining (BD Bioscience). All samples were assessed on a flow cytometer (Beckman, USA), and data were analyzed with FlowJo software (TreeStar).

### RNA-Seq

The nasal mucosa was collected on day 14 after the third immunization and homogenized with TRIzol reagent (Invitrogen, CA), and total RNA was isolated with chloroform/isopropanol, followed by purification with a RNeasy Mini Kit (Qiagen). For library and sequencing, the mRNA was isolated and purified from total RNA *via* Oligo(dT)-attached magnetic beads. Subsequently, purified mRNA was fragmented into small pieces with fragment buffer. Then first-strand cDNA was generated using random hexamer-primed reverse transcription, followed by a second-strand cDNA synthesis. Afterwards, A-Tailing Mix and RNA Index Adapters were added by incubating to end repair. The obtained cDNA fragments were amplified by PCR, and the products were purified by Ampure XP Beads and validated on the Agilent Technologies 2100 bioanalyzer for quality control. The double stranded PCR products were heated, denatured, and circularized by the splint oligo sequence to obtain the final library. The single strand circle DNA was formatted as the final library. The final library was amplified with phi29 to make DNA nanoballs (DNBs) which had more than 300 copies of one molecular, DNBs were loaded into the patterned nanoarray and pair end 100 bases reads were generated on BGIseq500 platform. The raw data were filtered by SOAPnuke software (35). The clean data were mapped on the reference Mus-musculus_GRCm38.p6 with hierarchical indexing for spliced alignment of transcripts (HISAT) software (36). Differentially expressed genes (DEGs) were identified and log2 transformed with DEseq2 (37). The resulting *p* values were adjusted to the Q value (adjusted p value) using the method of multiple testing adjustment with the R package (https://bioconductor.org/packages/release/bioc/html/qvalue.html). The DEGs identified according to the absolute value of log2(fold change) ≥1 and Q value (adjusted *P* value) ≤ 0.05. The enriched Gene Ontology (GO) and Kyoto Encyclopedia of Genes and Genomes (KEGG) pathways of the DEGs were analyzed by ClusterProfiler in RStudio. Heatmaps were drawn with the pheatmap R package.

### B. pertussis Challenge

Three weeks after the third immunization, all immunized mice were challenged with strain *B.p-L1 via* aerosol exposure using an aerosolization apparatus (38). Animals were infected *via* the challenge chamber for 30 min. Within the 30 min period, the air sample was removed from the sampling port at 5, 10, 20, and 30 min for assessment of the concentration of *B. pertussis* inside the chamber. The mock-infected animals received PBS aerosol exposure. At the indicated timepoints (2, 4, 7, 11, 16, and 23 days post infection), the lung, trachea, and nasal turbinate were harvested from mice to measure the bacterial burden (**Figure 1**).

### Quantitative Real-Time PCR (RT–qPCR)

Total RNA from lung tissues was extracted using TRIzol reagent (Invitrogen, USA) according to the manufacturer’s recommendations. Total RNA concentration and quality were measured by using a Thermo Scientific Varioskan Flash (Thermo Fisher Scientific, USA). cDNA was synthesized from 1 µg of RNA with oligo-dT primers and a PrimeScriptTM RT kit (Accurate Biotechnology, CHN). Cytokine mRNA levels were determined by RT–PCR performed on a LightCycler 96 system (Applied Biosystems, USA) using gene-specific primers (**Supplementary Table 1**) and a SYBR Green Premix Pro Taq HS qPCR Kit (Accurate Biotechnology, CHN). The expression of the housekeeping gene GAPDH was quantified in parallel for RNA normalization. The relative expression of the target genes was calculated by the ΔΔCt method.

### Histopathology

For histopathologic analysis, lung tissues from necropsied mice were fixed in 10% neutral buffered formalin, embedded in paraffin, and sectioned at 3-5 μm. Then, the sections were stained with hematoxylin and eosin (H&E) after dehydration. The pathological sections were observed and photographed under a microscope (Leica, Germany).

### Statistical Analysis

The results are presented as the means ± SEMs or GMTs and their 95% confidence intervals (CIs). For the data in accordance with normal distribution, one-way ANOVA followed by Tukey’s multiple comparisons test was used to compare the difference, while for the data not in accordance with normal distribution, Kruskal-Wallis followed by Dunn’s multiple comparisons test was used to compare the difference. A value of P < 0.05 was considered significant. Statistical analysis and plots were performed with the Prism version 8.0 (GraphPad Software, Inc.).

## Results

### c-di-GMP Can Better Promote BMDC Maturation Than Mammalian 2’,3’-cGAMP

**Figure 2:**
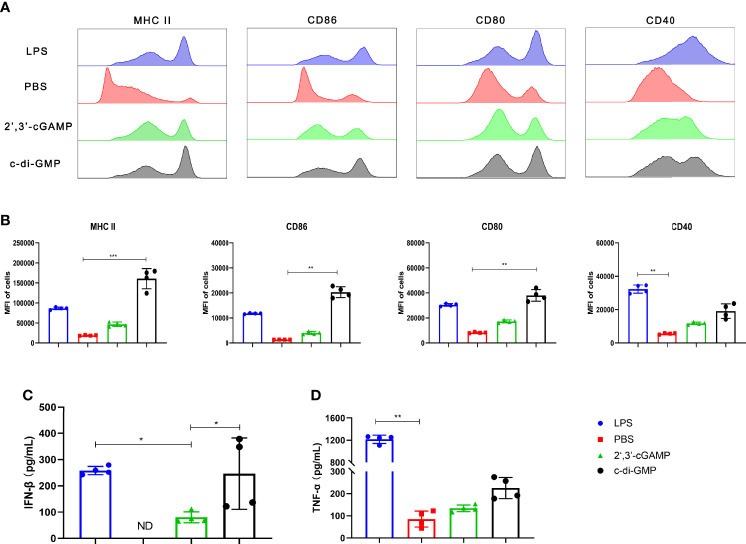
c-di-GMP can better promote BMDC maturation than mammalian 2’,3’-cGAMP. BMDCs were incubated with 2’,3’-cGAMP (5 μg/mL) or c-di-GMP (5 μg/mL) *in vitro* for 24 h. The cells were treated with lipopolysaccharide (LPS, 1 μg/mL) or sterile PBS as positive and negative controls, respectively. **(A, B)** BMDCs were collected and analyzed *via* flow cytometry to determine the surface expression of MHC II, CD86, CD80, and CD40. **(C, D)** Supernatants were collected, and the levels of IFN-β **(C)** and TNF-α **(D)** were analyzed. The data are expressed as the mean ± SEM of four independent experiments. “ND” indicates that no individuals in this group had detectable levels. The *P* value is indicated as follows: *P < 0.05, **P < 0.01, ***P < 0.001.

We studied the maturation efficacy of BMDCs by evaluating the BMDC maturation markers MHC II and the costimulatory molecules CD86, CD80, and CD40. In comparison, c-di-GMP treatment resulted in significantly higher expression of both MHC II and costimulatory molecules (CD86, CD80, and CD40) than 2’,3’-cGAMP, as revealed by the enhanced mean fluorescence intensity (MFI) (**Figures 2A, B**). Additionally, we measured the cytokine (IFN-β and TNF-α) levels secreted from BMDCs treated with LPS, PBS, 2’,3’-cGAMP, or c-di-GMP. The PBS-treated BMDCs showed undetectable amounts of IFN-β in the culture supernatant. Interestingly, compared with 2’,3’-cGAMP treatment, c-di-GMP treatment enhanced IFN-β secretion (**Figure 2C**). Moreover, compared with 2’,3’-cGAMP treatment, c-di-GMP treatment caused a slight increase in TNF-α production; however, the difference did not reach statistical significance (**Figure 2D**). Overall, these results indicated that c-di-GMP could better promote the production of the cytokines IFN-β and TNF-α and stimulate DC maturation than 2’,3’-cGAMP.

### The c-di-GMP-Adjuvanted aP Vaccine Elicits Robust Humoral and Mucosal Humoral Responses

To evaluate the systemic and mucosal humoral immune responses induced by different vaccines (**Table 1**), PT-, FHA-, and PRN-specific antibodies in serum, nasal washes (NW), and bronchoalveolar lavage fluid (BALF) were detected using ELISA method, and splenocytes and pneumonocytes producing antigen-specific IgA or IgG were detected using the ELISpot method at 14 days after the third immunization.

**Figure 3:**
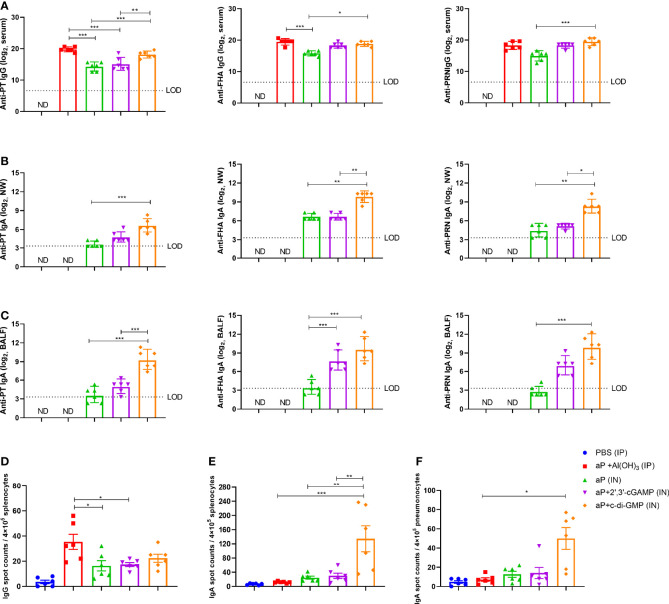
c-di-GMP elicits robust systemic and mucosal humoral responses to the acellular pertussis (aP) vaccine. Mice were intranasally administered the aP vaccine alone or adjuvanted with 2’,3’-cGAMP or c-di-GMP three times at three-week intervals and euthanized on day 14 after the last immunization. These groups were compared to a group that received a reference alum-adjuvanted aP vaccine *via* the intraperitoneal (IP) route three times at three-week intervals. Mice intranasally immunized with PBS served as the control group. ELISAs were used to compare antibody responses from mice immunized with different vaccines. ELISpot was conducted to assay the antibody-secreting splenocytes and pneumonocytes producing IgA or IgG against pertussis-specific antigens. **(A)** Total serum IgG titers against pertussis toxoid (PT), filamentous hemagglutinin (FHA), and pertactin (PRN) at day 14 after the last immunization. **(B)** Nasal wash (NW) IgA titers against PT, FHA, and PRN at day 14 after the last immunization. **(C)** Bronchoalveolar lavage fluid (BALF) IgA titers against PT, FHA, and PRN at day 14 after the last immunization. **(D)** Spleen tissues were harvested for the detection of PT-, FHA-, and PRN-specific IgG-secreting cells by ELISpot assay. **(E, F)** Spleen **(E)** and lung **(F)** tissues were assayed by ELISpot to assess PT-, FHA-, and PRN-specific IgA-secreting cells. Data are expressed as the mean ± SEM. The antibody results are expressed as the GMTs and their 95% confidence intervals (CIs). The dotted line indicates the limit of detection (LOD), and values that fell below the detection limit are represented by the limit of detection value for statistical analysis. “ND” indicates that no individuals in this group had detectable levels. Statistical differences between the results of vaccine-immunized groups and those of the PBS group are not marked. The *P* value is indicated as follows: *P < 0.05, **P < 0.01, ***P < 0.001.

In serum, the aP+c-di-GMP group vaccinated *via* IN administration (aP+c-di-GMP/IN) showed comparable levels of PT-, FHA-, and PRN-specific IgG as the aP+Al(OH)_3_ group vaccinated *via* IP administration (aP+Al(OH)_3_/IP), but both groups showed higher PT-specific IgG than the group vaccinated with aP vaccine without any adjuvant (aP/IN) or aP+2’,3’-cGAMP *via* IN immunization (aP+2’,3’-cGAMP/IN) (**Figure 3A**). In addition, the FHA- and PRN-specific IgG of the aP+c-di-GMP/IN group was not significantly different from that of the aP-2’,3’-cGAMP group but was significantly higher than that of the aP/IN group (**Figure 3A**). The aP+c-di-GMP/IN treatment also elicited higher serum PT-, FHA-, and PRN-specific IgA antibody levels than the aP+Al(OH)_3_/IP treatment and higher PRN-specific IgA antibody levels than the aP/IN treatment (**Supplementary Figure 1A**). Regarding antibody subclasses (IgG1 and IgG2a), aP+c-di-GMP/IN-immunized mice showed higher serum PT-specific IgG1 than aP/IN-immunized mice but comparable FHA- and PRN- specific IgG1 than aP+Al(OH)_3_/IP-immunized mice (**Supplementary Figures 1B–D**). In contrast, compared to aP+Al(OH)_3_/IP and aP/IN treatment, aP+c-di-GMP/IN treatment induced a significant increase in the levels of IgG2a against PT and PRN, and there was no significant difference in the levels of IgG2a against PT, FHA, and PRN between the two CDN-adjuvanted aP vaccine groups (**Supplementary Figures 1B–D**). These data suggested that the c-di-GMP-adjuvanted aP vaccine delivered *via* IN administration induced a balanced Th1 and Th2 immune response.

Regarding nasal washes (NW), aP+c-di-GMP/IN-vaccinated mice showed higher PT, FHA, and PRN-specific IgA levels than aP/IN-vaccinated mice and higher FHA and PRN-specific IgA levels than aP+2’,3’-cGAMP/IN-vaccinated mice, while there were no differences between the aP/IN and aP+2’,3’-cGAMP/IN groups (**Figure 3B**). Regarding BALF, aP+c-di-GMP/IN-vaccinated mice also showed the higher levels of IgA against PT, FHA, and PRN than aP/IN-vaccinated mice, but there was no significant difference in anti-PT, FHA, and PRN IgA levels in BALF between the two CDN-adjuvanted groups (**Figure 3C**).

For the antibody-secreting splenocytes and pneumonocytes producing IgA or IgG, the number of B cells secreting PT-, FHA-, and PRN-specific IgG in splenocytes from the alum-adjuvanted aP group was higher than that from the aP/IN and aP+2’,3’-cGAMP/IN groups, but it showed no difference from the aP+c-di-GMP/IN group (**Figure 3D**). However, in the pneumonocytes, both CDNs adjuvanted with aP vaccines produced a slight increase in the number of IgG-producing B cells (**Supplementary Figure 1E**). Of note, a higher frequency of B cells secreting anti-PT, anti-FHA, or anti-PRN IgA was observed in splenocytes from the aP+c-di-GMP/IN group than in those from all other groups, with a 4.5-fold higher frequency than in the aP+2’,3’-cGAMP/IN group (**Figure 3E**). And a higher frequency of B cells secreting anti-PT, anti-FHA, or anti-PRN IgA was also observed in pneumonocytes from the aP+c-di-GMP/IN group than in those from aP/IN group (**Figure 3F**). Although the frequency of B cells secreting anti-PT, anti-FHA, or anti-PRN IgA in pneumonocytes did not show statistical difference between the two CDN-adjuvanted groups, aP+c-di-GMP/IN-immunized mice caused a slight increase with 3.7-fold higher frequency than in the aP+2’,3’-cGAMP/IN group (**Figure 3F**). Taken together, these results suggest that aP+c-di-GMP treatment can elicit stronger systemic and mucosal humoral responses than 2’,3’-cGAMP.

### Intranasal Immunization With the c-di-GMP-Adjuvanted aP Vaccine Induces Strong Systemic and Mucosal Cellular Immune Responses and Potent Tissue-Resident Memory (TRM) Cells

**Figure 4:**
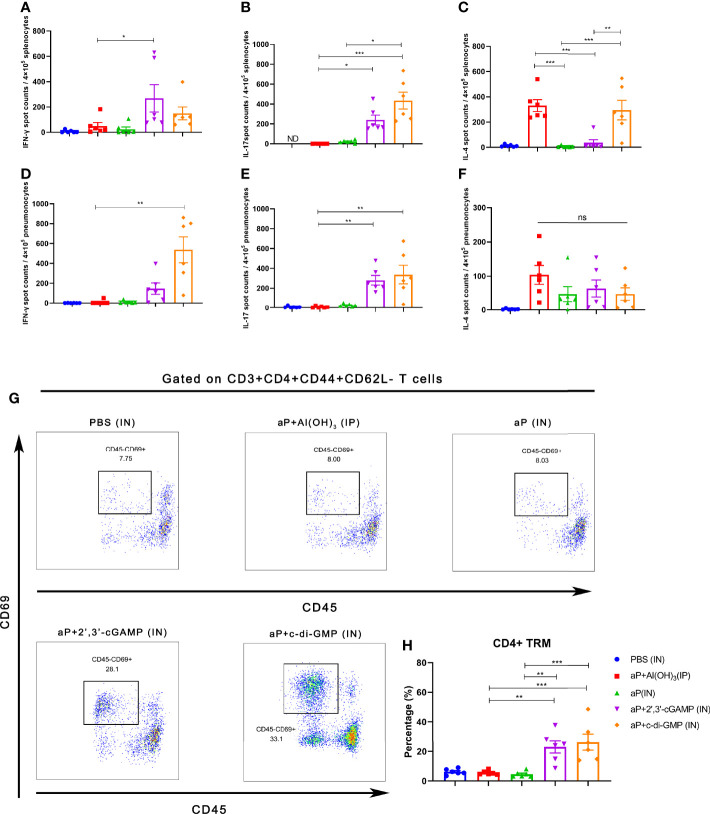
Intranasal immunization with a CDN-adjuvanted aP vaccine induces strong systemic and mucosal cellular immune responses and potent TRM cells. **(A–C)** Splenocytes were assayed by ELISpot assay for IFN-γ **(A)**, IL-17 **(B)**, and IL-4 **(C)** cell production after restimulation with the antigens PT (2 μg/mL), FHA (2 μg/mL), and PRN (2 μg/mL) (n=6 mice per group). **(D-F)** Lung tissues were assayed by ELISpot assay for cell production of IFN-γ **(D)**, IL-17 **(E)**, and IL-4 **(F)** after restimulation with the antigens PT (2 μg/ml), FHA (2 μg/ml), and PRN (2 μg/ml) (n=6 mice per group). **(G, H)** Mice were i.v. injected with an anti-CD45 antibody 10 min prior to euthanasia. The lungs were harvested, and lung mononuclear cells were stained with mAbs specific for CD3, CD4, CD44, CD62L, and CD69 for flow cytometric analysis. The results are expressed as CD4+ tissue resident cells (TRM): CD3+CD4+CD44+CD62L-CD45-CD69+; representative flow cytometry gating strategies for CD4+ TRM cells in the lungs are shown in **Supplementary Figure 2** (n=6 mice per group). Representative dot plots from flow cytometry analysis in **(G)** show the identification of CD4+ TRM cells. The relative proportion of CD4+ TRM cells in the lung tissues induced by different vaccines is shown in **(H)**. Data are expressed as the mean ± SEM. “ND” indicates that no individuals in this group had detectable levels. The *P* value is indicated as follows: *P < 0.05, **P < 0.01, ***P < 0.001, ns, no significance.

The *B. pertussis*-specific cellular immune responses were assessed *in vitro* by exposing splenic and pulmonary lymphocytes from immunized mice to the specific antigens PT, FHA, and PRN. Regarding frequencies of IFN-γ secreting T cells, aP+c-di-GMP/IN-treated mice showed increased frequency, but there was no significant difference compared to mice in other groups in splenic lymphocytes (**Figure 4A**), while the frequency was significantly higher than aP/IN-immunized mice, reaching a mean response of 542 spot-forming cells (SFC) per 4×10^5^ input splenocytes (**Figure 4D**). For frequencies of IL-17-secreting T cells, aP+c-di-GMP/IN-treated mice showed higher frequencies than mice in aP/IN and aP+Al(OH)_3_/IP groups and comparable frequencies to aP+2’,3’-cGAMP/IN-treated mice in splenic lymphocytes and comparable frequencies to aP+2’,3’-cGAMP/IN- and aP/IN-treated mice but higher than aP+Al(OH)_3_/IP- treated mice in pulmonary lymphocytes (**Figures 4B, E**). For IL-4-secreting T cells, the aP+c-di-GMP/IN group showed levels comparable to those in the aP+Al(OH)_3_/IP group but higher levels than the aP/IN and aP+2’,3’-cGAMP/IN groups in the spleen (**Figure 4C**), while the level increased but did not show significant differences from that in all other vaccine groups in pulmonary lymphocytes (**Figure 4F**).

To further examine whether TRM cells were induced by CDN-adjuvanted aP vaccines, flow cytometry was performed on lung tissues from mice (the TRM cell gating strategy is shown in **Supplementary Figure 2**). Not surprisingly, IP administration of the aP+Al(OH)_3_/IP vaccine did not generate an increased population of lung TRM cells (**Figures 4G, H**). IN immunization with aP vaccine alone also failed to promote the population of lung TRM cells (**Figures 4G, H**), indicating that mucosal immunization alone is not sufficient to induce lung TRM cells. In contrast, mice immunized with aP+2’,3’-cGAMP/IN or aP+c-di-GMP/IN showed significantly promotion of the population of lung TRMs (**Figures 4G, H**). Together, these data suggested that IN immunization with both CDN-adjuvanted aP vaccines promoted the generation of CD4+ TRM populations.

### The c-di-GMP-Adjuvanted aP Vaccine Generates Mixed Th1, Th2, and Th17 Responses

**Figure 5:**
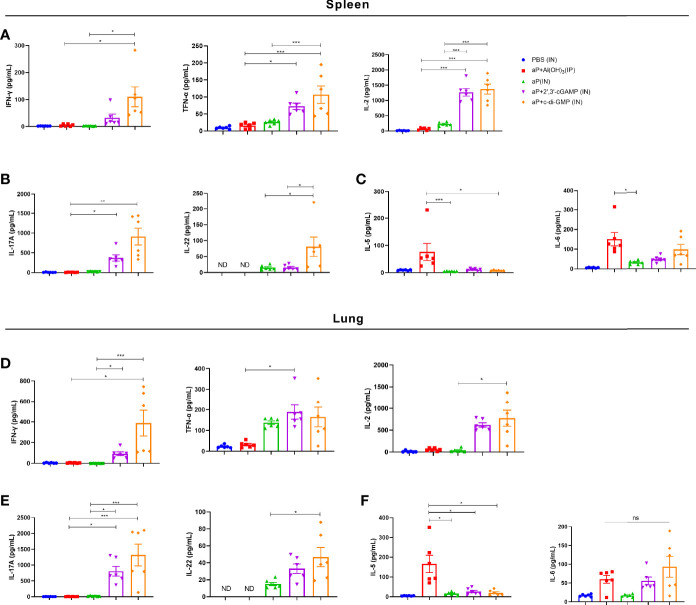
c-di-GMP-adjuvanted aP enhances the expression of Th1/Th17-related cytokines in both splenic and pulmonary lymphocytes. Two weeks after the third immunization, splenic and pulmonary lymphocytes were isolated. Cells were cultured at a concentration of 2 × 10^6^/ml at 37°C with 5% CO_2_ and stimulated with the *B. pertussis-*specific antigens PT (2 μg/mL), FHA (2 μg/mL), and PRN (2 μg/mL). After incubation for 3 days, the culture supernatant was collected, and multiple cytokines were assayed by ELISA. **(A)** Th1 response-associated cytokines (IFN-γ, TNF-α, and IL-2), **(B)** Th17 response-associated cytokines (IL-17A and IL-22), and **(C)** Th2 response-associated cytokines (IL-5 and IL-6) in the culture supernatant of splenic lymphocytes. **(D)** Th1 response-associated cytokines (IFN-γ, TNF-α, and IL-2), **(E)** Th17 response-associated cytokines (IL-17A and IL-22), and **(F)** Th2 response-associated cytokines (IL-5 and IL-6) in the culture supernatant of pulmonary lymphocytes. Data are expressed as the mean ± SEM. “ND” indicates that no individuals in this group had detectable levels. Statistical differences between the results of vaccine-immunized groups and those of the PBS group are not marked. The *P* value is indicated as follows: *P < 0.05, **P < 0.01, ***P < 0.001, ns, no significance (n=6).

After incubation with PT, FHA, and PRN for 3 days, cultures of splenic and pulmonary lymphocytes isolated after the third immunization were tested for cytokines in the supernatant. In culture supernatants of splenic lymphocytes, the levels of cytokines associated with Th1 responses (IFN-γ, TNF-α, and IL-2) and Th17 responses (IL-22) were significantly increased in the aP+c-di-GMP/IN group compared with those in the aP+Al(OH)_3_/IP or aP/IN group (**Figures 5A, B**). The aP+c-di-GMP/IN treatment also induced a slight increase in Th1 (IFN-γ, TNF-α, and IL-2) and Th17 (IL-17A and IL-22) related cytokine levels than aP+2′, 3′-cGAMP/IN treatment, but there was no significant difference (**Figures 5A, B**). Compared with aP/IN and aP+c-di-GMP/IN groups, the aP+Al(OH)_3_/IP group produced the higher IL-5, which is a Th2-related cytokine, in the supernatant of splenic lymphocyte cultures, while there was no significant difference in IL-6 levels between the aP+Al(OH)_3_/IP and aP+c-di-GMP/IN groups (**Figure 5C**).

In culture supernatants of pulmonary lymphocytes, similar to the results for splenic lymphocytes, compared with aP+Al(OH)_3_/IP or aP/IN treatment, aP+c-di-GMP/IN treatment greatly promoted the secretion of Th1 and Th17 response-related cytokines by pulmonary lymphocytes (**Figures 5D, E**). And aP+c-di-GMP/IN treatment also induced a slight increase in Th1 (IFN-γ, TNF-α, and IL-2) and Th17 (IL-17A and IL-22) related cytokine levels than aP+2′, 3′-cGAMP treatment, but there was no significant difference (**Figures 5D, E**). Similarly, the alum-adjuvanted aP vaccine induced higher IL-5 but not IL-6 levels than all other treatments, and the difference between the 2′, 3′-cGAMP/IN and aP+c-di-GMP/IN treatments was not significant (**Figure 5F**). Overall, these results indicated that IN delivering c-di-GMP-adjuvanted aP vaccine generates balanced Th1, Th2, and Th17 responses in both the spleen and lungs and that this effect is better than that with the 2′, 3′-cGAMP-adjuvanted aP vaccine.

### The c-di-GMP-Adjuvanted aP Vaccine-Immunized Mice Upregulated Th1, Th2, and Th17 Cell Differentiation and IgA Production Signaling in the Nasal Mucosa

**Figure 6:**
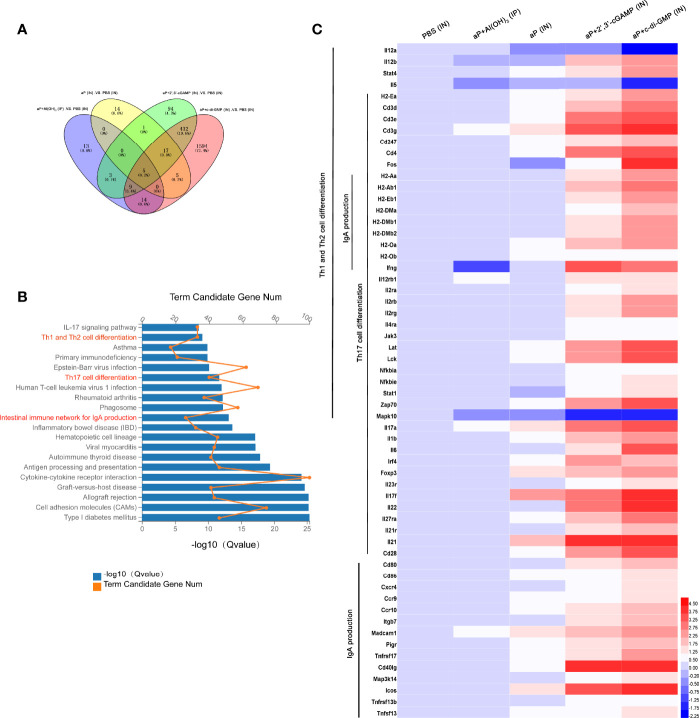
Differentially expressed genes (DEGs) in the nasal mucosa from c-di-GMP-adjuvanted aP immunized mice. **(A)** The DEGs for different vaccine-immunized mice. PBS-immunized mice at 14 days after the last immunization were used for comparison, and the overlap is shown as a Venn diagram. **(B)** The top 20 and KEGG pathways of the DEGs detected in the nasal mucosa of aP+c-di-GMP/IN- and PBS/IN-immunized mice are shown. **(C)** RNA-seq heatmap for the nasal mucosa from mice immunized with PBS/IN, aP+Al(OH)3/IP, aP/IN, aP+2’,3’-cGAMP/IN, or aP+c-di-GMP/IN (n = 5). The heatmap shows 59 significantly upregulated genes in aP+c-di-GMP/IN-immunized mice compared to those in other vaccine-immunized mice. Red (4.50) to blue (-2.25) were ranked by values of log2(value of gene expression).

RNA-seq analysis of the nasal mucosa in all immunized mice revealed 44 (7 upregulated, 37 downregulated), 42 (37 upregulated, 5 downregulated), 561 (407 upregulated, 154 downregulated), and 2076 (860 upregulated, 1216 downregulated) DEGs (|fold change| ≥2) in aP+Al(OH)_3_/IP-, aP/IN-, aP+2’,3’-cGAMP/IN-, and aP+c-di-GMP/IN-immunized mice, respectively, compared with PBS (IN)-immunized mice (**Figure 6A**).

The KEGG pathway functional enrichment results showed 20 top significant pathways (**Figure 6B**), among which Th1 and Th2 cell differentiation, Th17 cell differentiation, and IgA production signaling pathways were observed. Using a heatmap, 59 genes involved in these three signaling pathways were identified. Th1 and Th2 cell differentiation-, Th17 cell differentiation-, and IgA production signaling pathway-related genes were noticeably upregulated in aP+c-di-GMP-immunized mice (**Figure 6C**).

### The c-di-GMP-Adjuvanted aP Vaccine Reduces *B. pertussis* Burden in the Respiratory Tract

**Figure 7:**
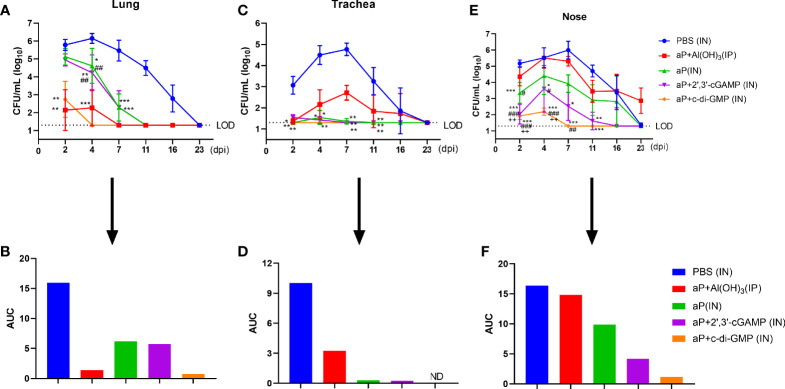
Intranasal administration of the c-di-GMP-adjuvanted aP vaccine reduces the respiratory *B. pertussis* burden. Mice were intranasally administered aP vaccine alone or adjuvanted with 2’,3’-cGAMP or c-di-GMP three times at three-week intervals and euthanized on day 14 after the last immunization. These groups were compared to a group that received a reference alum-adjuvanted aP vaccine *via* the intraperitoneal (IP) route three times at three-week intervals. Mice intranasally immunized with PBS served as the control group. Immunized mice were challenged by exposure to live *B. pertussis* three weeks after the third immunization. Analysis of bacterial burden was determined at 2, 4, 7, 11, 16, and 23 dpi. Bacteria were quantified by counting CFU from serial dilutions following challenge. **(A)** CFU counts were determined from lung homogenate. **(B)** The areas under the bacterial clearance curves corresponding to the curves of CFU counts from lung homogenate. **(C)** CFU counts were determined from tracheal homogenate. **(D)** The areas under the bacterial clearance curves corresponding to the curves of CFU counts from tracheal homogenate. **(E)** CFU counts were determined from nasal homogenate. **(F)** The areas under the bacterial clearance curves corresponding to the CFU counts from nasal homogenate. The results are the mean ± SEM (n = 5). The dashed line represents the lower limit of detection (LOD). ND” indicates that no individuals in this group had detectable levels. The *P* value is indicated as follows: *P < 0.05, **P < 0.01, ***P < 0.001 vs. PBS-immunized mice; ^#^*P <* 0.05, ^##^*P <* 0.01, ^###^*P <* 0.001 vs. alum-adjuvanted aP vaccine-immunized mice; and ++*P <* 0.01 vs. aP vaccine-immunized mice.

To assess protective efficacy against pertussis, immunized mice were challenged with aerosolized *B. pertussis*, and the bacterial colony-forming units (CFU) in nasal, tracheal, and lung homogenates were counted at the indicated times. aP+c-di-GMP/IN immunization provided a high level of protection against lung infection with *B. pertussis*, resulting undetectable bacterial colonization at 4 days post infection (dpi) with the lowest level of the areas under the bacterial clearance curve (AUC, 0.7195) among all groups (**Figures 7A, B**). When compared with those in PBS-immunized mice, the CFU counts in the lungs after *B. pertussis* aerosol challenge were significantly reduced in mice IP immunized with aP+Al(OH)_3_, resulting in undetectable CFU counts at 7 dpi and an AUC of 1.385, but the reduction was not as rapid as that seen in mice IN immunized with the aP+c-di-GMP vaccine (**Figures 7A, B**). Additionally, there was a significant reduction in the number of CFU in the lungs from aP (IN)- and aP+2’,3’-cGAMP (IN)-immunized mice at 4 dpi; however, these changes were not observed at 2 dpi, and the AUC of each was very similar (aP/IN: 6.209; aP+2’,3’-cGAMP/IN: 5.739) (**Figures 7A, B**).

In the trachea, *B. pertussis* colonization was not detected at any indicated time in the aP+di-GMP/IN group (**Figures 7C, D**). aP/IN- and aP+2’,3’-cGAMP/IN-immunized mice showed very low bacterial burdens at 2 dpi and 4 dpi, with AUCs of 0.30 and 0.24, respectively (**Figures 7C, D**). Surprisingly, aP+Al(OH)_3_/IP group mice did not exhibit detectable CFU counts in the trachea at 2 dpi; however, rebound was observed at 4 dpi, the numbers peaked at 7 dpi, and the bacteria were completely cleared at 23 dpi (**Figures 7C, D**).

Regarding nasal homogenate, PBS-immunized mice were heavily colonized at 2 days post *B. pertussis* challenge and reached the highest level, 1.0 × 10^6^ CFU/mL, at 7 dpi; the number of colonies gradually decreased until 23 dpi, with an AUC of 16.36 (**Figures 7E, F**). In contrast, mice immunized with aP+c-di-GMP/IN had very low CFU counts at 2 dpi and 4 dpi, and bacteria were completely cleared by day 7, with an AUC of 1.20 (**Figures 7E, F**). aP+2’,3’-cGAMP/IN treatment also resulted in protection in the nose, although not as effectively as that seen with aP+c-di-GMP/IN treatment, with an AUC of 4.191 (**Figures 7E, F**). Intriguingly, aP+Al(OH)_3_/IP treatment did not confer any protection against *B. pertussis* colonization of the mouse nose, resulting in a steady level of nasal colonization at 10^3^ CFU/mL through at least 23 dpi (**Figures 7E, F**). In contrast, the PBS-immunized mice completely cleared the nasal infection by that time. Together, these results indicated that IN immunization with the aP+c-di-GMP/IN vaccine enabled accelerated bacterial clearance in the respiratory tract following *B. pertussis* infection compared to that in aP+Al(OH)_3_/IP- or aP-2’,3’-cGAMP/IN-immunized animals.

### IN Immunization With the c-di-GMP-Adjuvanted aP Vaccine Protects Mice Against Lung Disease Caused by *B. pertussis* Infection

**Figure 8:**
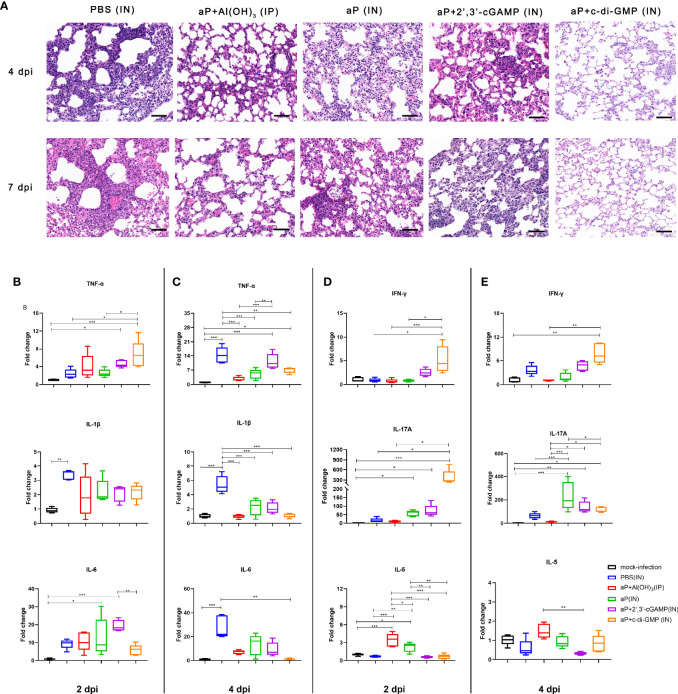
IN immunization with the c-di-GMP-adjuvanted aP vaccine protects mice against lung disease caused by *B. pertussis* infection. **(A)** Images showing H&E staining following infection with *B. pertussis*. The images shown are from 4 dpi and 7 dpi for all groups. Scale bar, 50 μm. Each image is representative of a group of 5 mice at 4 dpi and 7 dpi. **(B–E)** Total RNA was extracted from the lungs of mice euthanized at day 2 and day 4 after *B. pertussis* challenge. Mouse TNF-α, IL-1β, and IL-6 mRNA levels at 2 dpi **(B)** and 4 dpi **(C)** were quantified by RT–PCR. Mouse IFN-γ, IL-17A, and IL-5 mRNA levels at 2 dpi **(D)** and 4 dpi **(E)** were quantified by RT–PCR. GAPDH mRNA was used as an internal control. Data are shown as the fold change in gene expression compared to that in mock-infected animals (unimmunized and challenged with PBS) after normalization. n = 5 animals for each time point. Data are expressed as the mean ± SEM. The *P* value is indicated as follows: *P < 0.05, **P < 0.01, ***P < 0.001. See also **Supplementary Figure 3**.

We evaluated the pathological changes in the different vaccination groups after *B. pertussis* challenge. We observed lung histopathological changes by hematoxylin & eosin (H&E) staining at 4 and 7 dpi. Large amounts of inflammatory cell infiltration, especially neutrophils (blue arrows), lung interstitial thickening, and severe bronchial obstruction, were observed in the lungs of PBS-immunized mice after challenge (**Figure 8A**). A small quantity of inflammatory cells and relatively slight bronchial obstruction were also observed in lungs from aP/IN- or AP+2’,3’-cGAMP/IN-immunized mice (**Figure 8A**). Both aP+Al(OH)_3_/IP- and aP+c-di-GMP/IN-immunized mice showed less evidence of histopathological lesions in the lungs, especially no bronchial obstruction (**Figure 8A**). Interestingly, eosinophil infiltration was observed in mice IP immunized with the alum-adjuvanted aP vaccine, which was not found in other groups. And bronchial-associated lymphoid tissue (BALT) hyperplasia was observed in three groups of mice IN immunized vaccines, and with the c-di-GMP-adjuvanted aP vaccine most obviously, which was not found in control mice or mice IP immunized with the alum-adjuvanted aP vaccine (**Figure 8A** and **Supplementary Figure 3**).

Moreover, mRNA levels of cytokines in the lung tissues on 2 and 4 dpi were detected by RT-qPCR. The production of proinflammatory cytokines (IL-1β, TNF-α, and IL-6) in the lung tissues of *B. pertussis*-infected mice was expressed as a fold change compared to that in lung tissues from mock-infected mice (**Figure 8B**). At 2 dpi, PBS-immunized mice showed induction of only IL-1β levels in the lung tissues. However, the mRNA levels of IL-1β, TNF-α, and IL-6 were significantly increased at 4 dpi and were 14-, 5- and 28-fold higher than the levels in mock-infected lung tissues, respectively (**Figure 8C**). As expected, the levels of IL-1β, TNF-α, and IL-6 from mice immunized with aP+Al(OH)_3_/IP did not show obvious changes at 2 dpi and 4 dpi compared with those from mock-infected mice (**Figures 8B, C**). Interestingly, both CDN-adjuvanted groups showed higher TNF-α production than the mock-infected group at the two detected time points (**Figures 8B, C**). The difference was that the aP/IN and aP + 2’,3’-cGAMP/IN groups showed a slight increase and indistinguishable IL-6 production at 2 dpi compared with the mock-infected group, while no increase was observed in the aP+c-di-GMP/IN group (**Figures 8B, C**). We also analyzed the Th cell bias in the lungs of immunized mice at 2 dpi and 4 dpi by detecting the mRNA levels of IFN-γ, IL-17A, and IL-5, which are indicators of Th1, Th17, and Th2 activation, respectively. Mice immunized with aP+c-di-GMP/IN showed the higher levels of IL-17A and IFN-γ gene expression at 2 dpi than PBS- and aP+Al(OH)_3_/IP- immunized mice (**Figure 8D**). The aP/IN and aP+2’,3’-cGAMP/IN groups showed moderate upregulation of IL-17A gene expression at 2 dpi compared with that of the mock-infected group (**Figure 8D**). However, mice immunized with aP+Al(OH)_3_/IP did not show obvious changes in IL-17A and IFN-γ gene expression at 2 dpi and 4 dpi compared with that of mock-infected mice (**Figures 8D, E**), while they showed a significant increase in IL-5 expression at 2 dpi (**Figure 8D**).

## Discussion

Vaccination is important to control *B. pertussis* infection. However, pertussis has reemerged in recent years even with high vaccine coverage, which makes it remaining as a major global public health problem. Indeed, the high incidence of asymptomatic infection and the fact that current aP vaccines cannot prevent *B. pertussi*s transmission highlight the necessity to develop a more effective vaccine that can protect against disease and prevent *B. pertussis* infection and transmission (8–10). Considering that *B. pertussis* is a respiratory pathogen and highly contagious, efficacious prophylaxis would benefit from a mucosal immunization strategy to block bacterial replication at a very early stage and prevent *B. pertussis* infection and transmission. Here, we explored the potential of bacterial-derived c-di-GMP and mammalian 2′,3′-cGAMP as mucosal adjuvants for an aP vaccine against *B. pertussis*. Our results indicated that although both CDN adjuvants could produce in a certain reduction in *B. pertussis* burden in the URT compared with the aP vaccine alone administered *via* the IN route (aP/IN) or a conditional alum-adjuvanted aP vaccine administered *via* the IP route (aP +Al(OH)_3_/IP), the bacterial-derived c-di-GMP-adjuvanted aP vaccine (aP +c-di-GMP/IN) resulted in stronger systemic and local mucosal immune responses and better inhibited *B. pertussis* replication in both the upper and lower respiratory tract than the mammalian 2′, 3′-cGAMP-adjuvanted aP vaccine (aP+ 2′, 3′-cGAMP/IN).

Although CDN molecules have been explored as mucosal adjuvants, the immunostimulatory and immunoregulatory properties of different CDNs vary significantly (39, 40). Zhang et al. (27) demonstrated that 2′,3′-cGAMP induces stronger type I IFN production than other CDNs derived from bacteria. However, in the present study, we found that c-di-GMP elicited more IFN-β production in BMDCs than 2′,3′-cGAMP, resulting in stronger capacities to promote the activation and maturation of BMDCs. One of the reasons for this phenomenon may be related to different cell treatments. In a study by Zhang et al. (27), cells were permeabilized with digitonin, which allowed CDNs to more easily enter the intracellular space, where 2′,3′-cGAMP and STING possessed higher affinity than sting and other CDNs, resulting in more IFN-β production. In addition, in the current study, we found that c-di-GMP showed a better immunologic adjuvant effect for the aP vaccine than 2′, 3′-cGAMP *in vivo*. Blaauboer et al. (41) also found that c-di-GMP elicited higher antigen-specific antibody production, stronger T cell responses, and better protection against pneumococcal infection *in vivo* than mammalian 2′,3′-cGAMP. Indeed, CDNs, which were unable to penetrate the cell membrane and bind STING protein, enhanced antigen uptake *via* both pinocytosis and receptor-mediated endocytosis. However, the phosphodiester endogenic mammalian 2′,3′-cGAMP is unstable and more likely to be hydrolyzed by phosphodiesterases, especially ectonucleotide phosphodiesterase (ENPP1) of mammalian cells (42, 43). Thus, we considered that endogenous mammalian 2′,3′-cGAMP is more easily hydrolyzed by phosphodiesterases *in vivo*, which may result in its inferior adjuvant properties compared to bacterial-derived c-di-GMP. However, the specific mechanisms need to be further studied.

In the present study, aP+c-di-GMP/IN treatment significantly enhanced both systemic and mucosal immune responses and resulted in the fastest clearance of *B. pertussis* and an almost undetectable bacterial load in the respiratory tract among all vaccination regimens. In addition to strong IgG antibody responses in serum, strong mucosal humoral and Th17 responses should be considered important factors in bacterial clearance from the respiratory tract. In the current study, we found the highest level of IL-17-producing cells in the lung tissues in the aP+c-di-GMP/IN group after the last immunization, among all four vaccination groups. Moreover, we clearly identified significantly upregulated IL-17 expression in the lungs of aP+c-di-GMP/IN-immunized mice at 2 dpi. Substantial evidence has shown that Th17 and IL-17 play a pivotal role in protection against *B. pertussis* infection (13, 44, 45). Other intranasally delivered adjuvants, such as TLR agonists and combinations of TLR2 agonists and STING agonists, when delivered with *B. pertussis* antigens in mice, have all been shown to induce robust Th17 responses and confer *B. pertussis* control (14, 46). Thus, aP+c-di-GMP/IN inducing efficient Th17-related immune responses may contribute to resulting in rapid clearance of *B. pertussis* from the respiratory tract, with a lower bacterial load in the present study. More importantly, the aP+c-di-GMP/IN-immunized mice showed significantly higher IgA levels in NWs than the aP/IN- and aP+2’,3’-cGAMP/IN-immunized mice after the third immunization, which was also demonstrated by the upregulation of IgA production-associated genes in the nasal mucosa. Mucosal IgA is important to protect the nasal mucosa against *B. pertussis*. Solans et al. (47) found that BPZE1, the only mucosal aP vaccine candidate tested in clinical trials, induced protection in the nasal mucosa that was significantly diminished in IgA-deficient animals. Neither NW secretory IgA (sIgA) nor serum IgA from *B. pertussis* convalescent patients could inhibit adherence of *B. pertussis* to respiratory epithelial cells (48). In addition, sIgA production could be mediated by IL-17 (47), and IL-17 was central to protection against nasal infection with *B. pertussis* by recruiting neutrophils, especially siglec-F+ neutrophils (49). The RNA-seq results in this study suggested that the levels of many genes associated with Th17 proliferation were increased in the nasal mucosa of aP+c-di-GMP/IN mice, which may contribute to the clearance of bacteria from the nasal mucosa. Thus, we considered that the robust Th17 response and high level of IgA induced by aP+c-di-GMP/IN may contribute to the strong protection against *B. pertussis* in the entire respiratory tract, especially the URT. Third, Th1-related immune response elements, such as CD4+ T cells, may also contribute to immune protection during *B. pertussis* infection through IFN-γ-dependent mechanisms (45). We found a higher level of IFN-γ-producing cells in the lung tissues in the aP+c-di-GMP/IN-treated mice than in the aP+2’,3’ cGAMP-/IN-treated mice after the last immunization. Furthermore, we identified significantly upregulated IFN-γ expression in the lung in aP+c-di-GMP/IN mice 2 dpi. Evidence from both mouse models and clinical experiments has shown that cellular responses mediated by Th1 cells play an important role in protective immunity against *B. pertussis* and wP vaccine-mediated immune protection (44, 50–53). In addition, IFN-γ receptor knockout mice developed a disseminated lethal infection after challenge with *B. pertussis* (54). Moreover, many studies have shown that satisfactory protective results have been obtained *via* the addition of Th1-polarizing adjuvants to the existing aP vaccine (46, 55, 56). Thus, we considered that strong Th1 responses induced by aP+c-di-GMP/IN may also contribute to the clearance of bacteria from the airway.

One of the interesting results in this study was that although aP+c-di-GMP/IN provided the best protection for the lungs of the mice, with the fastest clearance of *B. pertussis*, followed by aP+Al(OH)_3_/IP, there was no significant difference between aP+2’,3’-cGAMP/IN and the nonadjuvanted aP vaccine administered *via* IN (aP/IN). In agreement with previous studies, mice immunized with aP+Al(OH)_3_/IP showed a small, limited amount of *B. pertussis* colonization and rapid clearance in the lungs after *B. pertussis* infection, causing only minor pathological damage to the lungs (8, 57). Compared with those from control mice or the three groups of IN immunized mice, lung tissue sections stained by HE from aP+Al(OH)_3_/IP mice showed that pulmonary neutrophilia was reduced, whereas eosinophilia was strongly increased, which we have rarely noticed previously. Moreover, IL-5 expression was significantly increased in the lung tissues of aP+Al(OH)_3_/IP-immunized mice after *B. pertussis* challenge. Verhoef et al. (58) found that IL-5 from type 2 innate lymphoid cells (ILC2s) in the lung could recruit eosinophils. In addition, clinical studies have demonstrated that eosinophil counts correlated with survival among patients with acute pulmonary infections (59, 60). Furthermore, a study from Linch et al. (61) highlighted that eosinophils possess strong antibacterial properties. Krishack et al. (62) revealed that eosinophilia was critical for the protection against *Staphylococcus aureus*, which further suggested a potential protective effect of eosinophilia against bacterial infection. Thus, we inferred that moderate infiltration of eosinophils in the lung tissues after *B. pertussis* challenge may contribute to the aP+Al(OH)_3_/IP protection mechanism, except for the high level of antibodies. Another interesting result in this study was that we observed a phenomenon similar to that in a study by Holubová et al. (57) in which an IP administered commercial alum-adjuvanted aP vaccine inhibited *B. pertussis* clearance from the nasal mucosa in a mouse model. In this study, mice given the aP+Al(OH)_3_/IP vaccine stabilized at a high level of *B. pertussis* colonization in NWs even at 23 dpi, with an average of 10^3^ CFU/mL. However, the control mice receiving only PBS had cleared the nasal bacteria by that time. Expectedly, mice IN immunized with the aP vaccine showed a lower bacterial load in the nasal mucosa than control mice, although both cleared nasal *B. pertussis* by 23 dpi. Indeed, Dubois et al. (63) also found that aP+Al(OH)_3_/IP immunization prolonged nasal carriage of *B. pertussis*. They further revealed the mechanism of this phenomenon in which the predominant Th2 immune response induced by aP+Al(OH)_3_/IP immunization may suppress the mucosal Th17 memory response, resulting in prolonged nasal carriage of *B. pertussis*. However, in contrast to the study of Boehm et al. (15), we found that IN delivery of an aP vaccine without mucosal adjuvant (aP/IN) did not elicit the same anti-PT IgG level, increased IL-17 level, or CD4+ TRM cell counts as aP+Al(OH)_3_/IP. Although aP/IN-immunized mice showed lower levels of specific IgA in NW and BALF samples and a reduction in bacterial load in the respiratory tract compared with those of control mice, they showed a lower bacterial load in the trachea and nasal mucosa but not in the lung tissue than aP+Al(OH)_3_/IP-immunized mice. This finding suggested that just simply modifying the aP vaccine immunization route, at least at the doses used in this study, did not accelerate the clearance of lung infection. In contrast, Boehm et al. (15) observed that an aP vaccine administered *via* the IN route induced protection of mouse lungs against *B. pertussis* comparable to that with the IP administered alum-adjuvanted aP vaccine. This discrepancy could be due to different doses of the aP vaccine used and different *B. pertussis* infection methods.

In summary, the obtained results showed that c-di-GMP, as a mucosal adjuvant, generated better antigen-specific antibody production and stronger Th1 and Th17 responses than the mammalian cyclic dinucleotide 2′3′-cGAMP. This difference translated into better protection against *B. pertussis* infection in a mouse model, with more efficient bacterial clearance in the respiratory tract. These results also indicated that c-di-GMP may be a better candidate mucosal adjuvant for regulating immune responses.

## Data Availability Statement 

The datasets presented in this study can be found in online repositories. The names of the repository/repositories and accession number(s) can be found below: https://www.ncbi.nlm.nih.gov/bioproject/?term=PRJNA806201.

## Ethics Statement

The animal study was reviewed and approved by Committee on Ethics of the Institute of Medical Biology, Chinese Academy of Medical Sciences.

## Author Contributions

LS and MS conceived and designed the research. WJ, XW, and YS performed experiments. and data analysis. LC and JYL analyzed the data. JLL and QG contributed reagents and materials. WJ wrote the manuscript. LS and MS reviewed the manuscript. All authors contributed to the article and approved the submitted version.

## Funding

This work was supported by the by the Yunnan Provincial Science and Technology Department [Grant number 202002AA100009]; and the Kunming Science and Technology Bureau [Grant number 2019-1-N-25318000003332]; and the Special Funds for High-Level Health Talents of Yunnan Province [Grant number L-201615]. The funders had no role in the design of the study, data collection and analysis, decision to publish, or preparation of the manuscript.

## Conflict of Interest

The authors declare that the research was conducted in the absence of any commercial or financial relationships that could be construed as a potential conflict of interest.

## Publisher’s Note

All claims expressed in this article are solely those of the authors and do not necessarily represent those of their affiliated organizations, or those of the publisher, the editors and the reviewers. Any product that may be evaluated in this article, or claim that may be made by its manufacturer, is not guaranteed or endorsed by the publisher.
