# Supplementary material for: Intranasal Immunization With a c-di-GMP-Adjuvanted Acellular Pertussis Vaccine Provides Superior Immunity Against Bordetella pertussis in a Mouse Model
Source: Front Immunol. 2022 Apr 13;13:878832. doi: 10.3389/fimmu.2022.878832 (PMC9043693; doi:10.3389/fimmu.2022.878832)
Supplement: Supplementary file 1 [file DataSheet_1.pdf]

Supplementary Material

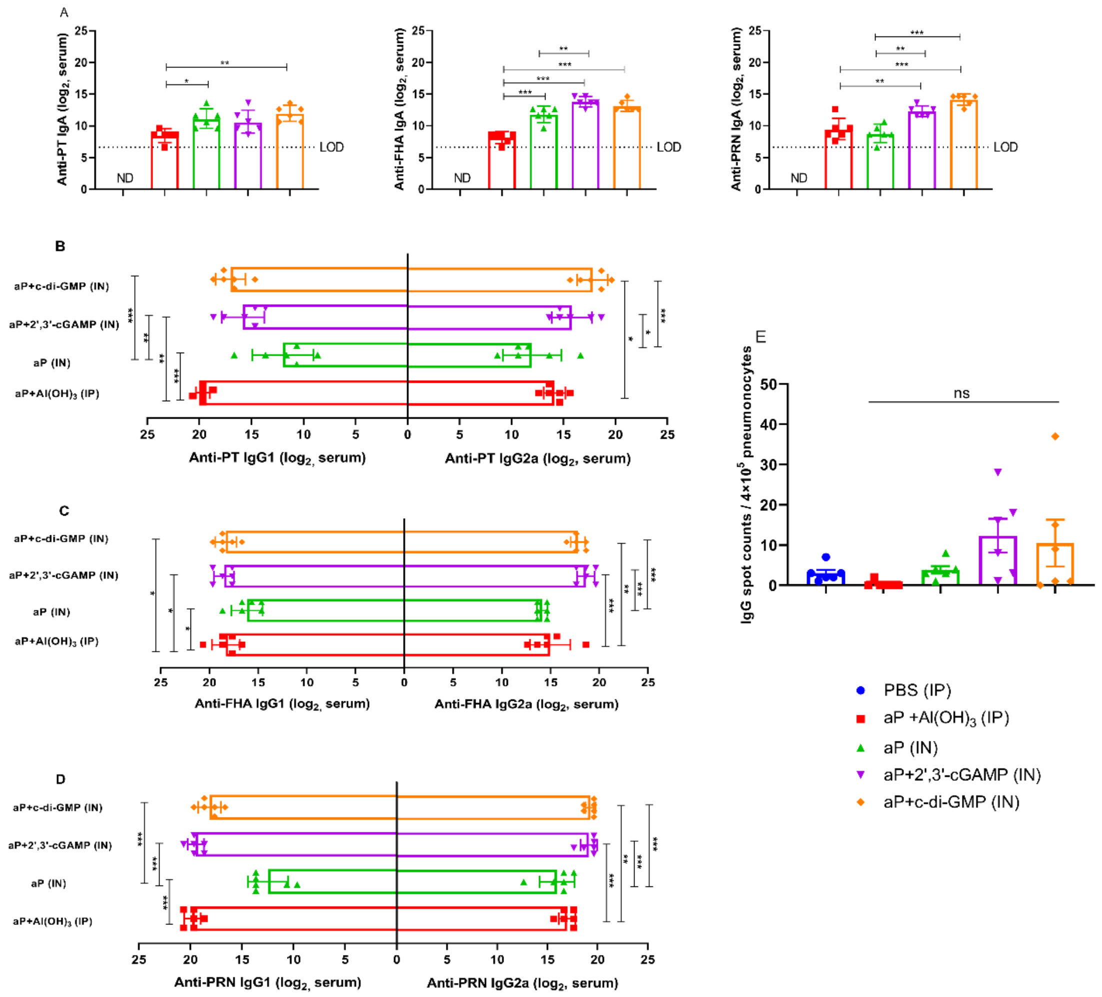

Supplementary Figure 1. c-di-GMP elicits robust systemic and mucosal humoral responses to the acellular pertussis (aP) vaccine. BALB/c mice were immunized as indicated for Figure 1. Serum was collected two weeks after the last immunization, and antibody isotypes were analyzed. (A) Serum IgA specific for PT, FHA, and PRN was assayed by ELISA. (C-D) IgG1 and IgG2a specific for PT (B), FHA (C), and PRN (D). (E) Spleen tissues were assayed by ELISpot to assess PT-, FHA-, and PRN-specific IgG-secreting cells. Data are expressed as the mean ± SEM. The results for antibody levels are reported as GMTs and their 95% confidence intervals (CIs). The dotted line indicates the limit of detection (LOD), and values that fell below the detection limit are represented by the limit of detection value for statistical analysis. “ND” indicates that no individuals in this group had detectable levels. Statistical differences between the results for vaccine-immunized groups and the PBS group are not marked. Significance was determined via one-way ANOVA with a

Tukey multiple comparison test. The P value is indicated as follows: *P <0.05, **P < 0.01, ***P< 0.001, ns: no significance.

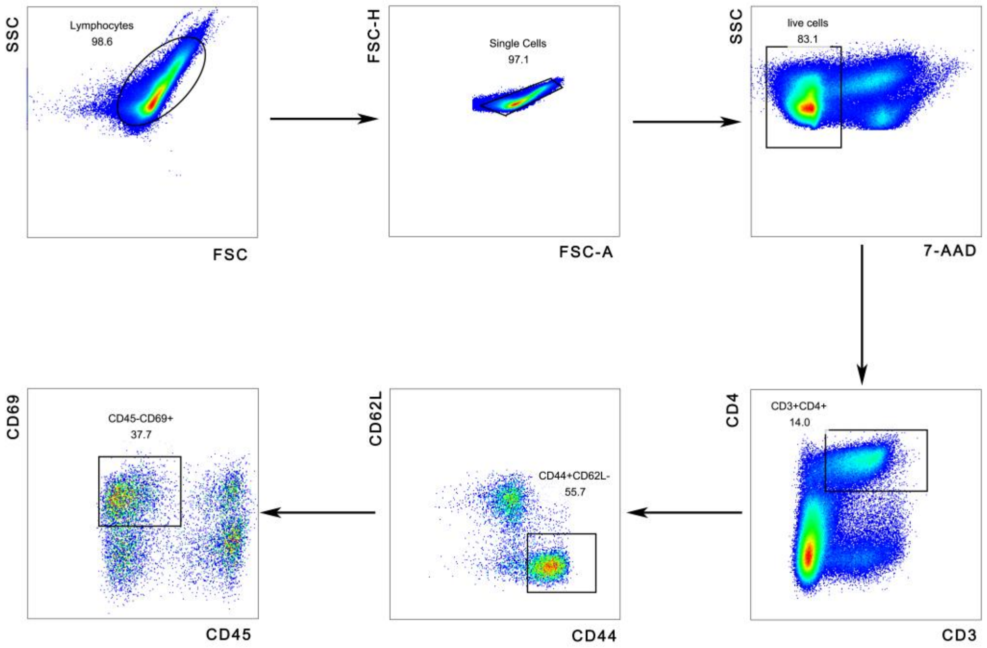

Supplementary Figure 2. Gating strategy used to isolate CD3+CD4+ CD44+CD62L-CD69+ T cells. Live single lymphocytes were gated based on 7-AAD staining. T cells were identified based on CD3+ and CD4+ expression. CD3+CD4+CD44+CD62L- cells were gated based on the marker expression patterns of CD44+ and CD62L-. Then, CD3+CD4+CD44+CD62L- cells were gated based on CD45RA; CD45RA- cells represented lung tissue-resident cells, and CD45+ cells represented

cells in the peripheral circulation. Finally, CD3+CD4+CD44+CD62L-CD45- cells were gated based on the expression of the tissue homing marker CD69. These data are shown in Figure 4G and F.

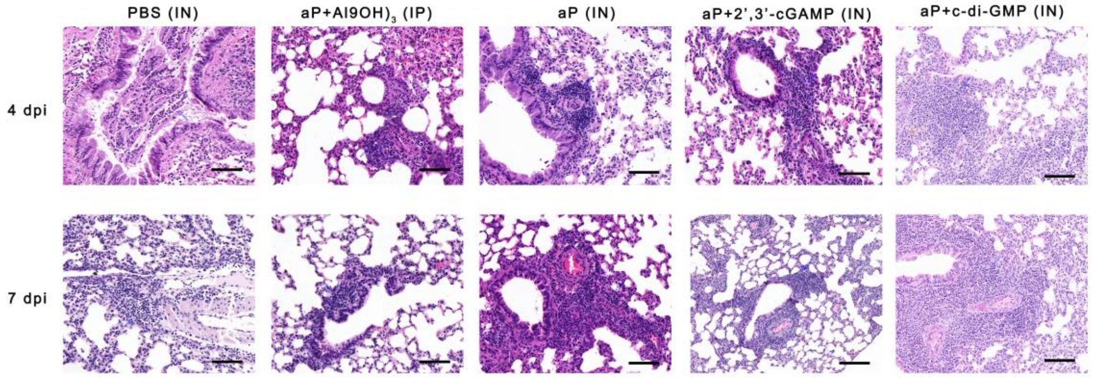

Figure S3. H &E staining for the lung tissues of mice infected with *B.pertussis*. Images show H&E staining for the lung tissues following infection with *B.pertussis*. Scale bar, 50 µm. Each image is representative of a group of 5 mice for 4 dpi and 7 dpi.

Supplemental Table 1. Primer sequences of each primer used

Primers	Sequence (5’-3’)
GAPDH forward	AGGTCGGTGTGAACGGATTTG
GAPDH reverse	GGGGTCGTTGATGGCAACA
TNF-α forward	CCACGTCGTAGCAAACCACC
TNF-α reverse	CGGCTGGCACCACTAGTTG
IL-1β forward	TCTATACCTGTCCTGTGTAATG
IL-1β reverse	GCTTGTGCTCTGCTTGTG
IL-6 forward	CTGCAAGAGACTTCCATCCAG
IL-6 reverse	AGTGGTATAGACAGGTCTGTTGG
IFN-γ forward	GGAGGAACTGGCAAAAGGATG
IFN-γ reverse	GACGCTTATGTTGTTGCTGATGG
IL-17A forward	TTTAACTCCCTTGGCGCAAAA
IL-17A reverse	CTTTCCCTCCGCATTGACAC
IL-5 forward	CTCTGTTGACAAGCAATGAGACG
IL-5 reverse	TCTTCAGTATGTCTAGCCCCTG
